# Modelling human-to-human transmission of monkeypox

**DOI:** 10.2471/BLT.19.242347

**Published:** 2020-07-08

**Authors:** Rebecca Grant, Liem-Binh Luong Nguyen, Romulus Breban

**Affiliations:** a Institut Pasteur, Emerging Diseases Epidemiology Unit, 25-28 rue du Dr. Roux, 75015 Paris, France.

Monkeypox is an emerging infectious disease for which outbreak frequency and expected outbreak size in human populations have steadily increased.^1^ The geographic spread of monkeypox cases has expanded beyond the forests of central Africa, where cases were initially found, to other parts of the world, where cases have been imported. This transmission pattern is likely due to the worldwide decline in *orthopoxvirus* immunity, following cessation of smallpox vaccination, once smallpox was declared eradicated in 1980. Monkeypox could therefore emerge as the most important *orthopoxvirus* infection in humans.^2^ We use mathematical modelling to argue that, in a population with diminishing herd immunity against *orthopoxvirus* species, the epidemic potential of monkeypox will continue increasing.

Monkeypox is caused by the monkeypox virus, member of the *orthopoxvirus* genus in the Poxviridae family. This genus includes three other human pathogens: variola virus (causing smallpox), cowpox virus and vaccinia virus. Monkeypox and smallpox yield similar clinical presentations, with monkeypox causing lymphadenopathy, as a distinguishing feature, early in the disease course.^2^ Smallpox infection leads to long-lasting immunity; repeat attack rates of smallpox are just about 1 in 1000 for 15–20 years.^3^ Smallpox vaccination with vaccinia, a first-generation vaccine, also yields long-lasting immunity, with an efficacy of 80–95%. The current recommendation for revaccination is every 10 years, although longitudinal studies suggest that protection may last much longer.^4^ Vaccinia is also known to deliver long-lasting immunity against monkeypox, with 85% efficacy.^5^ Furthermore, studies of antibody responses to *orthopoxvirus* species suggest perfect cross-immunity between smallpox and monkeypox.

No concurrent epidemics of smallpox and monkeypox have ever been reported. Smallpox is known to be a human-only disease, while monkeypox is a zoonotic disease, whereby introductions in human populations take place from a currently unidentified animal reservoir. In particular, contacts with animal species in forests of western and central Africa, notably Central African Republic, Democratic Republic of the Congo, Nigeria and Republic of the Congo, result in sporadic monkeypox introductions into human populations. All monkeypox outbreaks were self-limiting, with human transmission chains ending without establishing epidemics.^2^ With the eradication of smallpox, monkeypox appears to emerge as the dominant pox disease in humans. Currently, the epidemic risk for humans is considered to be small.^6^

The reproduction number, denoted by R, is often used to quantify the ability of an emerging disease to invade a population. R is defined as the expected number of secondary infections per primary infection, whereby the basic reproduction number, R_0_, refers to the context of a fully susceptible population. When R is above 1, epidemic potential has been reached. An R_0_ above 1 indicates that the disease has epidemic potential. Mathematical modelling of smallpox transmission^7^ often invokes the simplifying assumption that smallpox infection or vaccination yields perfect, lifelong immunity. The same assumption may hold for monkeypox, for which much less is known about infection. The theory of transmission of infectious diseases with perfect cross-immunity shows that, between two infectious diseases competing to infect susceptible hosts, the disease with the larger R_0_ prevails, while the other is eliminated. The R_0_ for smallpox has previously been estimated between 3.5 and 6.0;^7^ hence, theoretically, R_0_ for monkeypox must be smaller. Furthermore, the theory emphasizes the critical role of the animal reservoir for the persistence of monkeypox. In the case where herd immunity is induced through vaccination at birth, with a vaccine delivering perfect, lifelong immunity, which only takes in a fraction *ε* of those vaccinated (*ε* is called vaccine efficacy), R and R_0_ are related by the following equation: R = R_0_ (1-*εp*), where *p* is the vaccination coverage and *εp* is the effective vaccination coverage.

We performed analyses of the transmission potential for both smallpox and monkeypox, using data collected in the Democratic Republic of the Congo during 1966–1984. Smallpox vaccination in this country ended in 1980, with vaccination coverage of nearly 100%. Assuming that R_0_ for smallpox was 3.5–6.0,^7^ and vaccine efficacy was 80–95%,^3^ we estimated R for smallpox at 0.59 (uncertainty bounds 0.18–1.2), consistent with the epidemiological observation of no smallpox cases in the country beyond 1980. Data collected in the Democratic Republic of the Congo during 1980–1984^5^^,^^8^ show that R for the Congo basin clade of monkeypox at that time was 0.32 (uncertainty bounds 0.22–0.40).^9^ This result is consistent with the epidemiological observation that monkeypox transmission among humans in the country was not self-sustained.^5^^,^^8^ However, using 85% for vaccinia efficacy (meaning effective coverage) against monkeypox^1^ and the above equation, we calculated R_0_ for monkeypox to be 2.13 (uncertainty bounds 1.46–2.67), smaller than R_0_ for smallpox, but larger than 1. We therefore postulate that monkeypox had epidemic potential in the Democratic Republic of the Congo in the early 1980s.

**Fig. 1:**
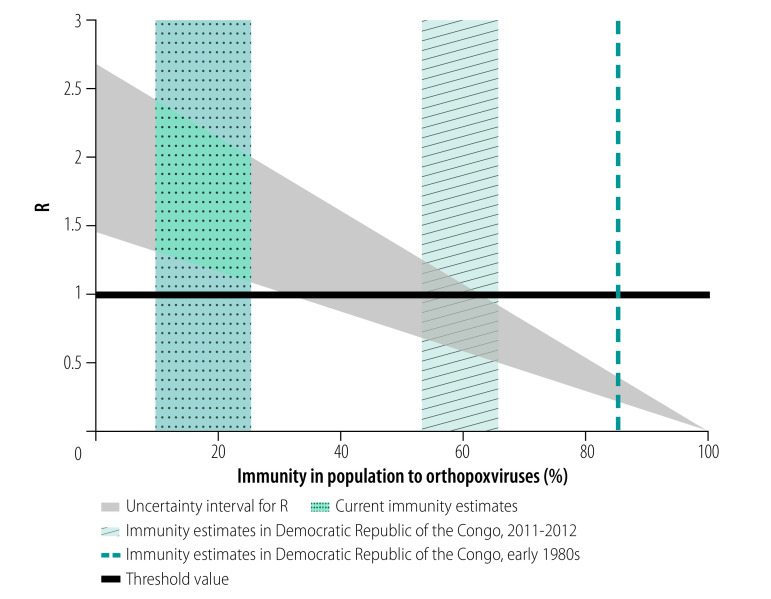
The reproduction number R for monkeypox as function of immunity in a population to *orthopoxvirus* species

According to our model, a monkeypox epidemic could not have started in this country between 1980 and 1984, since 85% of the population was immune to monkeypox; monkeypox was observed as a rare disease in humans, occurring only sporadically. Since then, the Democratic Republic of the Congo has reported increased monkeypox human infections^1^ and parts of the country have been declared monkeypox-endemic areas.^1^^,^^2^ In 2011–2012, the population immunity against *orthopoxvirus* species was only 60% (95% confidence interval, CI: 53–65%);^10^ that is, 96% (95% CI: 91–99%) among individuals vaccinated against smallpox and 26% (95% CI: 18–35%) among individuals unvaccinated against smallpox,^10^ which suggests regular contact of the country’s population with *orthopoxvirus* species. Using this immunity data, we recalculated R to be 0.85 (uncertainty bounds 0.51–1.25). The estimates of R for monkeypox in the Democratic Republic of the Congo are illustrated in Fig. 1. The theory proposes two scenarios of how monkeypox could be endemic in this country. First, frequent outbreaks, with R < 1, may occur due to involuntary human contact with the animal reservoir hosting monkeypox. Second, monkeypox may undergo sustained human-to-human transmission (R > 1). In either case, repeated circulation of monkeypox in human hosts, particularly immunocompromised hosts, favours pathogen evolution and emergence of newly human-adapted pathogens, depending on R and on the human pathogen fitness landscape.^11^

R: reproduction number; UI: uncertainty interval.

Notes: The R estimates for the monkeypox epidemiology in the Democratic Republic of the Congo in the early 1980s when the smallpox vaccination campaign was coming to an end. The current estimates of R represent countries where accidental exposure to *orthopoxvirus* species may be considered negligible.

Several caveats are in order, as our analyses rely mostly on data from the Democratic Republic of the Congo. R_0_ estimates may not be readily generalizable, as they depend on virus clade, society structure and organization, and population density. R estimates also depend on the level of the immunity in the population. Our analyses are intended for homogeneous populations and are, thus, conservative. For example, segregation by age for daily activity can yield segregation by *orthopoxvirus* immunity status, which can only exacerbate societal frailty to monkeypox invasion. Still, crude estimates^7^ show that residual *orthopoxvirus* immunity, in countries where natural exposure to *orthopoxvirus* species is negligible, may have already fallen in the range of 10–25%, which corresponds to R in the range of 1.10–2.40 (Fig. 1). This value suggests that monkeypox could establish itself as an endemic disease in such settings, starting from imported human or animal cases.

The clinical presentation of monkeypox facilitates outbreak investigations around incidentally imported cases. The incubation period of monkeypox is 5–21 days, followed by clinical onsets for up to 21 days. Monkeypox is not considered contagious during its incubation period and asymptomatic monkeypox infection has not been documented. Transmission occurs through fluids secretion, mainly from the respiratory tract or skin lesions. The distinctive symptoms of human monkeypox greatly aid in its early detection and containment. Nevertheless, secondary transmission from imported cases is possible, as evidenced by the case of nosocomial transmission to a health-care worker in the United Kingdom of Great Britain and Northern Ireland in 2018.^12^ Under stringent infection prevention and control measures, including case isolation, hand hygiene, use of personal protective equipment to avoid direct contact with patients and the use of standard, contact and droplet precautions, the likelihood that an imported case triggers an epidemic can be expected to be low. Yet, with increasing importation rate, monkeypox outbreak investigations may become a costly and poorly effective strategy, to prevent endemic disease.

We conclude that circulation of smallpox, followed by worldwide smallpox vaccination, have previously protected human populations from monkeypox epidemics. We combined historical data on smallpox and monkeypox with mathematical modelling to estimate the basic reproduction number of monkeypox, and found that monkeypox has epidemic potential. This finding may explain the increasing number of monkeypox outbreak reports, resulting in endemic monkeypox in central African countries. Moreover, with declining immunity to *orthopoxvirus* species, monkeypox can pose an ever-increasing threat for health security.
